# Extracellular Sulfatases, Elements of the Wnt Signaling Pathway, Positively Regulate Growth and Tumorigenicity of Human Pancreatic Cancer Cells

**DOI:** 10.1371/journal.pone.0000392

**Published:** 2007-04-25

**Authors:** Roman Nawroth, Annemieke van Zante, Sara Cervantes, Michael McManus, Matthias Hebrok, Steven D. Rosen

**Affiliations:** 1 Department of Anatomy, University of California San Francisco, San Francisco, California, United States of America; 2 Department of Pathology, University of California San Francisco, San Francisco, California, United States of America; 3 Diabetes Center, University of California San Francisco, San Francisco, California, United States of America; 4 Comprehensive Cancer Center, University of California San Francisco, San Francisco, California, United States of America; Ordway Research Institute, Inc., United States of America

## Abstract

**Background:**

Heparan sulfate proteoglycans (HSPGs) are control elements in Wnt signaling, which bind extracellularly to Wnt ligands and regulate their ability to interact with signal transduction receptors on the cell surface. Sulf-1 and Sulf-2 are novel extracellular sulfatases that act on internal glucosamine-6-sulfate (6S) modifications within HSPGs and thereby modulate HSPG interactions with various signaling molecules, including Wnt ligands. Emerging evidence indicates the importance of reactivated Wnt signaling in a number of cancers, including pancreatic adenocarcinoma.

**Principle Findings:**

Both Sulf proteins were upregulated in human pancreatic adenocarcinoma tumors and were broadly expressed in human pancreatic adenocarcinoma cell lines. Expression of human extracellular sulfatases Sulf-1 and Sulf-2 enhanced Wnt signaling in a reconstituted system. Three of four pancreatic adenocarcinoma cell lines tested exhibited autocrine Wnt signaling, in that extracellular Wnt ligands were required to initiate downstream Wnt signaling. Exposure of these pancreatic adenocarcinoma cells to a catalytically inactive form of Sulf-2 or siRNA-mediated silencing of endogenous Sulf-2 inhibited both Wnt signaling and cell growth. Sulf-2 silencing in two of these lines resulted in markedly reduced tumorigenesis in immunocompromised mice.

**Conclusions/Significance:**

We have identified the Sulfs as potentiators of autocrine Wnt signaling in pancreatic cancer cells and have demonstrated their contribution to the growth and tumorigenicity of these cells. Since the Sulfs are extracellular enzymes, they would be attractive targets for therapy of pancreatic cancer. Our results run counter to the prevailing view in the literature that the Sulfs are negative regulators of tumorigenesis.

## Introduction

Pancreatic adenocarcinoma is the most common form of pancreatic cancer and one of the most deadly malignancies with a 5 year survival rate of 3% and a median survival of less than 6 months [1]. There has been recent interest in the role of embryonic signaling pathways in cancer. Considerable evidence has demonstrated that these pathways remain functional in a limited number of cells within the adult and that dysregulation of these pathways contributes to the formation and persistence of tumors [2], [3]. In this regard, the reactivation of Notch, Hedgehog and Wnt pathways have attracted recent interest in cancers of the GI tract, including pancreatic adenocarcinoma [2], [4], [5].

The dysregulation of Wnt signaling in pancreatic adenocarcinoma is the focus of the present study. Wnts comprise a large family of secreted proteins, which regulate cell growth, apoptosis, motility and differentiation during embryonic development [3]. In brief, activation of the canonical Wnt pathway is initiated by binding of Wnt ligands to signal transduction receptors on the cell surface, which leads to the accumulation of non-phosphorylated β-catenin in the cytoplasm. After its translocation to the nucleus, β-catenin forms a complex with members of the TCF/LEF family of transcription factors, and activates target genes [3].

Wnt signaling was first implicated in cancer through the analysis of mammary gland tumorigenesis induced by the mouse mammary tumor virus (MMTV) [6]. A high proportion of the tumors were due to activation of Wnt1 expression through insertion of the MMTV provirus into the *wnt1* gene. Tumorigenesis is thought to be based on an autocrine Wnt transforming pathway in which Wnt ligand expression by mammary epithelial cells provides a growth stimulus to the same cells. Recently autocrine Wnt signaling was demonstrated in breast cancer, ovarian cancer and myeloma cell lines, [7], [8]. This mechanism, in which extracellular Wnt ligands provide an essential stimulus for cell proliferation, is distinct from that commonly found in human colon cancer and several other forms of cancer, in which constitutive activation of Wnt signaling is a consequence of mutations in downstream cytoplasmic elements such as *APC, CTNN1* (encoding β-catenin) or *AXIN2* [3].

These characteristic mutations in downstream Wnt signaling molecules are very rare in pancreatic adenocarcinoma [9]. Yet, aberrant activation of Wnt signaling is a significant feature of human pancreatic adenocarcinoma, as revealed by the nuclear localization of β-catenin in a significant fraction (30–65%) of tumors [9], [10]. Consistent with the histochemical evidence, biochemical analysis has established a marked elevation of total β-catenin and its increased nuclear localization in two-thirds of pancreatic adenocarcinomas [9]. Moreover, in mechanistic studies with pancreatic adenocarcinoma cell lines, inhibition of Wnt signaling resulted in reduced cell proliferation and survival in vitro [11].

An area of current interest are the extracellular regulatory elements in the Wnt signaling pathway. Among these are secreted Wnt antagonists such as Frizzled related proteins (sFRP), Wnt inhibitory factor (WIF) and the Dickkopf family (DKK) [12]. Downregulation of any of these antagonists initiates Wnt activation and contributes to several forms of cancer [12]. A class of extracellular proteins that positively regulates Wnt activity are heparan sulfate proteoglycans (HSPGs). Glypicans, for example, are required for Wnt signaling during development in Drosophila and vertebrates [13]. HSPGs are also necessary in examples of Wnt-dependent tumor formation [14], [15]. The myriad functions of HSPGs in growth and differentiation of cells [16] are mediated through their ability to bind to a diverse repertoire of growth factors and morphogens. Binding depends on the sulfation pattern of glucosamine (N-, 3-O, and 6-O positions) and uronic acid (2-O-position) within the attached heparan sulfate chains. [16]. 6-O-sulfation (6S) of glucosamine is of particular importance in the binding of many important ligands to HPSGs, including the Wnts [17]–[19]. A newly appreciated mechanism for regulating signaling by these ligands is by post-synthetic “editing” of the 6S pattern of HSPGs through the action of a class of extracellular sulfatases known as the Sulfs [20].

QSulf-1, the first member of the Sulf family to be described, was discovered in an analysis of muscle development in the quail embryo [20]. This was followed by the description of its orthologues in rat [21], mouse, and human [22] and the discovery of the closely-related homologue, Sulf-2 [22]. The Sulfs are neutral pH optimum endosulfatases that remove 6-O-sulfate from internal glucosamine residues in highly sulfated subdomains of heparin/HSPGs [18], [19], [22], [23]. The initial studies of QSulf-1 revealed a positive role in the regulation of Wnt signaling during muscle specification in the quail embryo [20]. This activity depends on the ability of QSulf-1 to reduce the interaction of Wnt ligands with HSPGs. The authors proposed a two state model in which HS chains form a high affinity HS-Wnt complex, which sequesters Wnt from interacting with its signal transduction receptors. The affinity of Wnt for HS is weakened when QSulf-1 modifies the 6S pattern of the HS chains, allowing the initiation of Wnt signaling [19].

Sulf-dependent promotion of Wnt signaling has also been observed in other developmental events [24]. In vitro evidence suggests that the Sulfs can promote other signaling pathways including angiogenesis and BMP signaling [23], [25]. Importantly, *sulf* null mice show reduced body mass [26], [27]. This phenotype is consistent with positive regulatory roles for the Sulfs during normal development.

The discovery that canonical Wnt signaling is activated in pancreatic adenocarcinoma (see above) has focused our attention on the possible involvement of the Sulfs in this cancer. In the present study, we demonstrate upregulation of both Sulf proteins in pancreatic adenocarcinoma tumors. Using human pancreatic adenocarcinoma cancer cell lines, we establish a role for Sulf-2 in promoting autocrine Wnt signaling, in vitro cell growth and tumorigenicity. This positive contribution of a Sulf to Wnt-dependent tumor growth is in striking contrast to several reports in which the enforced expression of Sulfs antagonizes other signaling pathways (e.g. FGF-2, and HGF) in tumor cells and suppresses cell growth [28]–[34].

## Results

### Sulf-1 and Sulf-2 are upregulated in human pancreatic cancer

Mining of public DNA microarray datasets and a quantitative PCR study establish that *SULF1* transcripts are upregulated in human pancreatic cancer (Table 1). The latter study found that the mean level of *SULF1* mRNA was 22.5-fold greater in the cancer tissue (n = 31) than in normal pancreas (n = 19) [31]. *In situ* hybridization localized expression of *SULF1* to tubular structures, likely representing invasive carcinoma.

**Table 1:** *SULF1* mRNA expression in human pancreatic cancers

Study	Comparison	Result
Li et al., 2005 [31]	normal pancreas (19 specimen) vs. pancreatic cancer (31 specimen)	•22 of 31 cancer specimens had higher *SULF1* mRNA levels than in highest normal. mean *SULF1* mRNA level was 22.5-fold higher in cancer vs. normal
Su et al., 2001 [50]	pancreatic adenocarcinoma (6 specimens)	•5/6 samples showed upregulation of *SULF1* mRNA relative to its mean level in 100 primary tumors of 11 different kinds (p = 0.039)
Iacobuzio-Donahu et al., 2003 [39] (www.oncomine.org)	normal pancreas (5) vs. pancreatic adenocarcinoma (12)	•*SULF1* mRNA level in adenocarcinomas was 2.8 std deviations above that in normal pancreas (p = 5.5×10^−6^)

**Figure 1:**
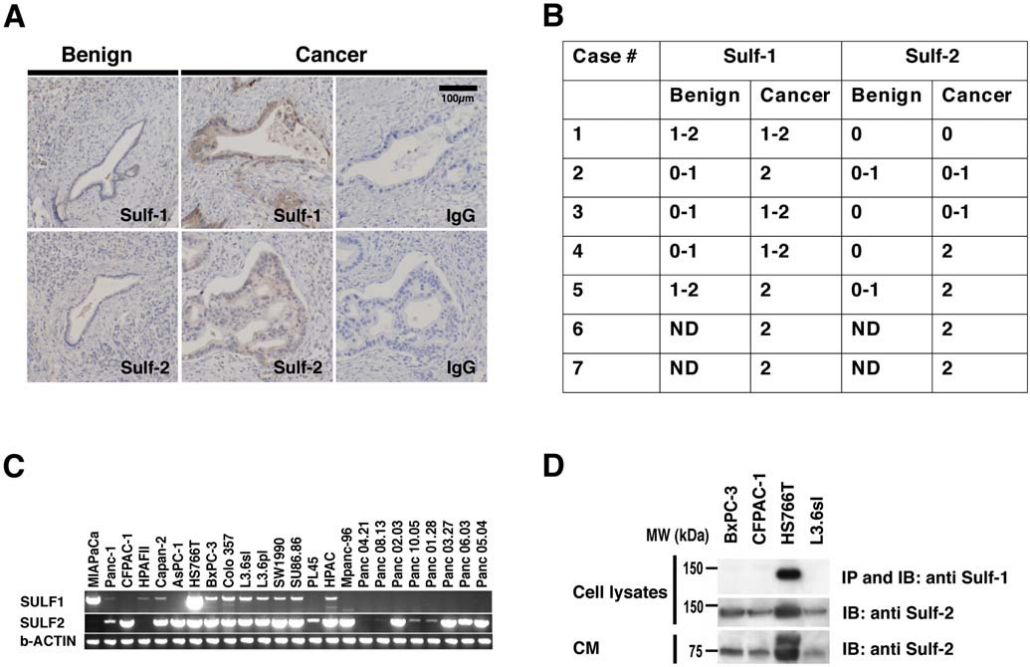
Detection of Sulf expression in pancreatic adenocarcinoma cell lines and tumors. A: Representative serial sections of pancreatic adenocarcinoma were stained with Sulf-1, Sulf-2 and control IgG. Benign ducts were compared to areas of invasive carcinoma on the same section. B: Quantitative tabulation of Sulf immunohistochemistry results in pancreatic adenocarcinoma cases (0 = no or trace staining,1 = moderate staining,2 = strong staining, ND = not determined). C: RNA was isolated from 24 adenocarcinoma cell lines, cDNA was prepared, and subjected to PCR with primers for *SULF1* and *SULF2* and β-*ACTIN* as loading control. D: Immunoblots were performed on cell lysates and conditioned medium (CM) using antibodies against Sulf-1 (upper panel) and Sulf-2 (middle and lower panel). Only Sulf-2 protein was detected in the CM.

To determine if Sulf proteins were expressed by pancreatic adenocarcinoma in surgical specimens, we stained sections from seven archived cases with Sulf-1 and Sulf-2 antibodies and an IgG control (Figure 1 A and B). Benign interlobular ducts on the same tissue sections as the invasive carcinoma served as an internal control. In 7/7 cases, the malignant epithelial cells generally showed moderate to strong staining for Sulf-1 (Figure 1B). For Sulf-2, 4/7 cases showed strong staining of these cells, 2/7 cases had moderate staining and 1 case was negative. Most benign ducts showed no staining or only trace staining for Sulf-1 (≈54%) or Sulf-2 (≈88%). Focal staining for Sulf-1 or Sulf-2 was seen in a minority of the benign ducts. Diffuse weak staining of fibroblasts for both Sulfs was observed in some areas of stroma, usually in association with inflammatory cells. There was no staining with the IgG control.

Next, we determined expression of the Sulfs in human pancreatic adenocarcinoma cell lines by using RT-PCR to survey a panel of 24 cell lines. These were derived from either primary or metastatic pancreatic adenocarcinomas [5]. *SULF1* transcripts were detected by RT-PCR in 12/24 and *SULF2* transcripts in 21/24 of these lines (Figure 1C). To examine expression at the protein level, we investigated 4 of these cell lines, 3 of which were positive for both *SULF* transcripts (CFPAC-1, HS766T, L3.6sl) and 1 which was positive for only *SULF2* (BxPC-3). We performed immunoblots on detergent lysates and conditioned medium (CM) derived from these cells (Figure 1D). Sulf-1 protein was detected in only one cell line (HS766T cells), whereas Sulf-2 protein was present in all 4. In detergent lysates, the molecular weight of the major species was 130 kDa, which corresponds to an unprocessed form for both proteins [22]. Sulf-2 protein was released into CM by all 4 cell lines. In contrast, Sulf-1 was not detected in HS766T CM. As previously observed for several breast carcinoma cell lines [25], Sulf-2 protein was detected in CM as a processed 75 kDa component.

### Sulf-1 and Sulf-2 promote Wnt signaling

QSulf-1 has been shown to promote activation of Wnt signaling pathways in response to exogenous Wnt ligands [20]. We chose to study the effects of the human Sulfs on Wnt signaling in a similar reconstituted system [20]. We overexpressed the Sulfs in HEK 293T and co-cultured these cells with fibroblasts that expressed either Wnt1 or Wnt4. Wnt signaling activity was measured by the TCF-luciferase reporter system (TOPflash/FOPflash), a standard method for measuring β-catenin-dependent transcriptional activation [20]. Expression of each Sulf augmented Wnt signaling in response to either Wnt ligand, whereas mock-transfected HEK 293T cells did not respond (supplementary figure, Fig. S1). Thus, both HSulfs, like QSulf-1 [20], can promote Wnt signaling.

### Expression of sFRP-2 inhibits Wnt signaling in pancreatic adenocarcinoma cell lines

**Figure 2:**
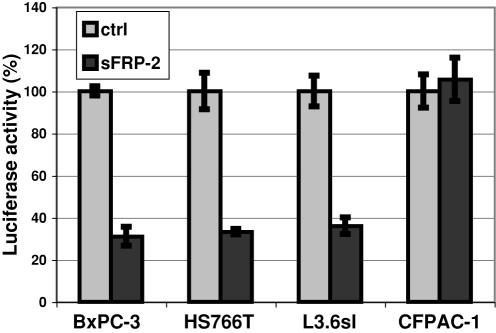
Autocrine Wnt signaling in pancreatic adenocarcinoma cell lines. The indicated pancreatic adenocarcinoma cell lines were transfected with sFRP-2 cDNA (sFRP-2) or control vector (ctrl) and the TOP/FOPflash reporter system. Wnt activity was determined by luciferase activity. The values shown are the means±SD's for 3 independent determinations. Differences between sFRP and control were significant in the Student t-test with p values of<0.002 for BxPC-3, HS766T and L3.6sl.

Previous work has established active Wnt signaling within a series of the pancreatic adenocarcinoma cell lines, including the 4 lines above [M. Pasca di Magliano and M. Hebrok, unpublished observations]. To determine whether Wnt signaling in these 4 lines was autocrine in nature, we employed secreted Frizzled-related protein-2 (sFRP-2) as an extracellular Wnt antagonist [35], [36]. We transiently transfected the cell lines with a cDNA for sFRP-2 or with the empty vector (ctrl) and measured Wnt activity by the TCF-luciferase reporter assay. sFRP-2 expression diminished Wnt signaling ≈60% in 3 of the lines but had no effect on CFPAC-1 cells (Figure 2). These results establish that the BxPC-3, HS766T and L3.6sl cells possess an autocrine Wnt signaling pathway.

### Catalytically inactive human sulfatase protein inhibits Wnt signaling and cell growth

**Figure 3:**
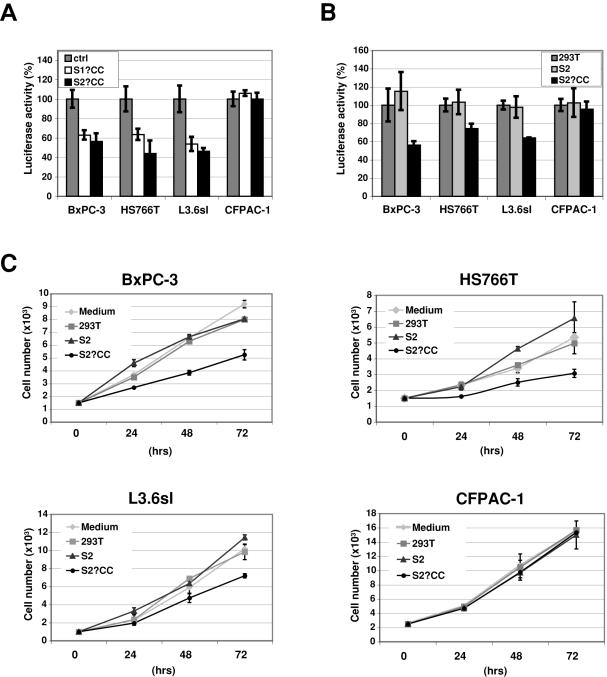
The effects of inactive Sulfs on Wnt signaling on Wnt signaling and cell growth. **A:** Four pancreatic adenocarcinoma cell lines as indicated were transiently transfected with cDNAs encoding either a catalytically inactive form of Sulf-1 (“S1ΔCC”) or a catalytically inactive form of Sulf-2 (“S2ΔCC”) or with the empty vector (“control”). The mutants were made by replacing two adjacent cysteines with alanines. Wnt signaling was determined by utilizing the TOP/FOPflash reporter system. The values shown are the means±SD's for 4 independent determinations and differences between mutant and control cells were statistically significant with p values<0.02 for BxPC-3, HS766T and L3.6sl cells (Student t-test). **B:** The pancreatic adenocarcinoma cell lines were cultured with conditioned medium derived from each of the following: parental HEK-293 cells (“control”), HEK 293T cells stably expressing wild-type Sulf-2 (denoted “Sulf-2”), or expressing a catalytically inactive form of Sulf-2 (“S2ΔCC”). Wild-type Sulf-2 protein and the mutant were present in the conditioned medium of the two producing cells at approximately equivalent levels. Wnt signaling was determined as before with the TCF-luciferase reporter assay. The values shown are the means±SD's for 3 independent determinations and differences between Sulf-2 mutant and control were statistically significant for BxPC-3 (p = 0.002), HS766T (p = 0.01) and L3.6sl (p = 9×10^−6^) (Student t-test). **C:** The pancreatic adenocarcinoma cell lines were cultured in a transwell system over feeder layers consisting of: medium alone (“Medium”); parental HEK 293T cells (“HEK 293T”); HEK 293T cells stably producing wild-type Sulf-2 (“Sulf-2”); or a catalytically inactive form of Sulf-2 (“S2ΔCC”). Cell growth was monitored by hemacytometer counting for 4 days. The values shown are the means±SD's for 3 independent determinations.

We next asked if the endogenous Sulfs in these cell lines were directly involved in Wnt signaling. When we overexpressed the Sulfs, there was no effect on Wnt signaling (data not shown). Since all of these cells expressed significant levels of the Sulfs, we next asked whether there might be inhibitory effects if we expressed catalytically inactive forms of these proteins. We have previously shown that mutation of two cysteines in the sulfatase domain of each Sulf eliminated sulfatase activity [22]. We transfected a cDNA encoding inactive Sulf-1 (S1ΔCC), Sulf-2 (S2ΔCC) or empty vector (ctrl) into each of the 4 cell lines and again measured Wnt activity. As shown in Figure 3A, expression of either inactive Sulf inhibited Wnt activity ≈50% in all but CFPAC-1 cells. The 3 responding lines were the same that exhibited autocrine Wnt signaling (Figure 2). The fact that either inactive Sulf exerted equivalent effects on these pancreatic cancer cell lines suggests functional redundancy of the two enzymes.

We next asked whether the exogenous addition of catalytically inactive Sulf protein would also interfere with Wnt signaling. As a source of Sulf-2 protein, we used HEK 293T cells which were stably transfected either with wild-type human Sulf-2 (S2) or catalytically inactivated Sulf-2 (S2ΔCC) [25]. We also used the parental HEK 293T cells (293T) as controls. The stable transfectants secreted the active and inactive Sulfs at comparable levels (data not shown). We incubated the pancreatic adenocarcinoma cells for 16 hours with CM from these cells or with control medium and compared Wnt signaling. Exposure to the inactive Sulf-2 protein (S2ΔCC) inhibited Wnt signaling in the same 3 cell lines that were responsive to transfection with the S2ΔCC cDNA (Figure 3B), whereas CFPAC cells did not show an effect. The addition of medium, conditioned with active Sulf-2 protein, did not promote Wnt signaling over the basal level.

Next, we set up a co-culture system to examine the effects of recombinant Sulf protein on cell growth of the same cell lines. The pancreatic cancer cells were seeded at low density on transwell filters above feeder layers, which consisted of parental HEK 293T cells or HEK 293T expressing active or inactive Sulf-2. Cell counts were monitored for the next several days. The results paralleled the suppressive effect we observed on Wnt signaling. Thus, as compared to control medium, S2ΔCC CM inhibited the growth of BxPC-3, HS766T and L3.6sl cells (Figure 3C) but had no effect on CFPAC-1 cells. In contrast, active Sulf-2 did not promote or inhibit cell growth of any of the lines.

### Lentiviral shRNA silencing of Sulf-2 in pancreatic adenocarcinoma cells reduces Wnt signaling, cell proliferation, and cell survival

**Figure 4:**
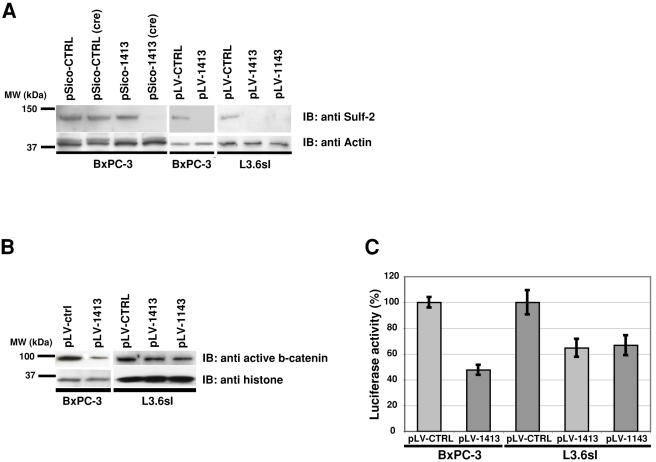
Wnt signaling in cells silenced for Sulf-2. **A:** Cells were transduced with the conditional (pSico) or nonconditional lentivirus (pLVTHM) encoding either Sulf-2 specific (1413, 1143) or control (ctrl) shRNAs. The cells were lysed and equal amounts of proteins were subjected to SDS-PAGE. In case of the pSico, cells were lysed 10 days after cre transfection. Sulf-2 protein levels were determined by performing immunoblots and the same lysates were probed with anti actin antibody as a loading control. B: 100 µg total protein of the nuclear fraction from the cells were applied to SDS-PAGE and amounts of active beta-catenin were detected using an antibody against non-phosphorylated beta-catenin. As loading control, the same lysates were analyzed with an anti histone antibody. C: Parental, pLV-ctrl, pLV-1413 and pLV-1143 transduced cells were transfected with the FOP/TOPflash reporter system and luciferase activity was detected 48 hrs later. The values shown are the means±SD's for 3 independent determinations and differences between ctrl and Sulf-2 silenced cells were statistically significant (BxPC-3, p = 0.002 and (L3.6sl, p = 0.013 and 0.017 for the two silencing vectors).

To further investigate the role of Sulf-2 in Wnt signaling and cell growth, we used shRNA methodology to silence the expression of Sulf-2 in these cells. We employed three shRNA constructs: two independent constructs targeting Sulf-2 (1413 and 1143) and a control (ctrl). These constructs were used in different vector backgrounds in which the presence or absence of GFP allowed us to monitor expression of the shRNAs (see **MATERIALS AND METHODS**). Transduced cells were sorted for GFP expression and subsequently analyzed for Sulf-2 expression. The 1143 and 1413 constructs (pLV-1143, pLV-1413) produced 80% and 90% reductions of Sulf-2 protein, respectively, in L3.6sl cells, relative to the control shRNA (pLV-ctrl) (Figure 4A). There was no reduction of the protein in the pLV-ctrl treated cells relative to untreated cells (data not shown). The pLV-1413 construct produced a similar reduction in BxPC3 cells in two different vector backgrounds (pLV and pSico).

Inactivation of Wnt signaling results in decreased levels of activated β-catenin (N-terminally non-phosphorylated) in the nucleus [3]. We therefore isolated the nuclear fraction of Sulf-2 silenced and control cells (BxPC-3 and L3.6sl) and analyzed levels of activated β-catenin. There was a ≈80% decrease of β-catenin in Sulf-2 silenced BxPC-3 cells, and a ≈30% reduction in L3.6sl cells (Figure 4B). We also quantified Wnt activity using the TCF-luciferase reporter system. Consistent with the β-catenin result, this assay confirmed that Wnt signaling was substantially inhibited in the Sulf-2 silenced cells relative to control-treated cells for both BxPC-3 and L3.6sl cells (Figure 4C). Again, BxPC-3 cells showed stronger inhibition (52% with pLV-1413) than the L3.6sl cells (35% inhibition with pLV-1413 or pLV-1143).

**Figure 5:**
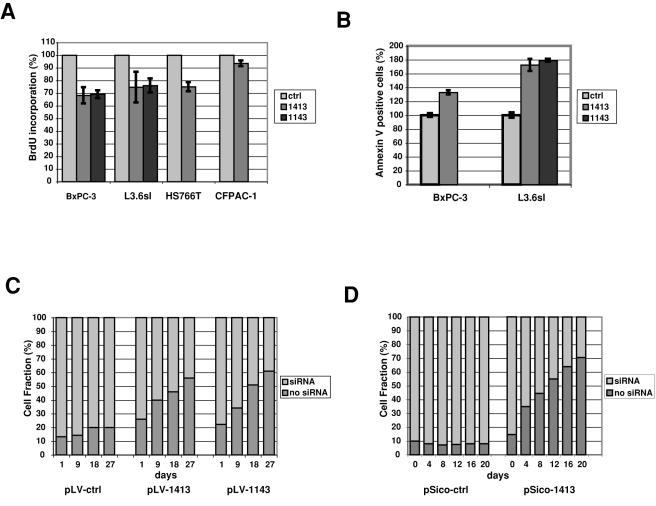
The effects of Sulf-2 siRNAs on proliferation, apoptosis, and growth of pancreatic cancer cells. **A:**The indicated cells were transduced with control (pLV-ctrl) or Sulf-2 shRNA (pLV-1413, pLV-1143). The cells were then incubated with BrdU for one hour and BrdU incorporation was detected by flow cytometry analysis. The values shown are the mean +/- SD's for 2 independent experiments. The differences in BrdU uptake between ctrl and Sulf-2 silenced cells were statistically significant for BxPC-3 (p<0.005), L3.6sl (p<0.007) and HS766T (p<0.001). B: BxPC-3 and L3.6sl pancreatic cells were transduced with control (pLV-ctrl) or Sulf-2 shRNA (pLV-1413, pLV-1143) were harvested and incubated with PE-conjugated annexin V. Subsequently cells were subjected to flow cytometry analysis. The differences between between ctrl and Sulf-2 silenced cells were statistically significant with p-values of 0.0001 for BxPC-3 and p<0.004 for L3.6sl. C and D: L3.6sl cells were transduced with either pLV-1413, pLV-1143 or pLV-ctrl and mixed populations of siRNA and non-siRNA expressing cells were co-cultured. Ratios of transduced to non-transduced cells were measured over time by GFP expression. BxPC-3 cells were transduced with either pSico-ctrl or pSico-1413 and transiently transfected with cre-recombinase to activate expression of the shRNA. The resulting mixed populations of cells with activated (GFP-) and inactivated (GFP+) shRNA expression were co-cultured. Ratios of GFP+ to GFP- cells, using flow cytometry were analyzed as a function of time. Expression of GFP did not have any effect on cell growth in the control population.

We determined the effects of Sulf-2 silencing on cell proliferation by measuring the incorporation of 5-bromodeoxyuridine (BrdU) into the cells. There was a 25–30% reduction of cells undergoing S-phase in L3.6sl, BxPC3, and HS766T cells, and only a 5% reduction in CFPAC-1 cells (Figure 5A). Additionally, we performed apoptosis assays by measuring annexin V staining. The Sulf-2 silenced BxPC-3 cells showed an increase in apoptosis of 33%, whereas the L3.6sl cells showed increases of 65% and 79% with two independent shRNAs (Figure 5B).

To examine cell growth over time in Sulf-2 silenced cells, we compared growth of cells in which Sulf-2 was silenced with non-transduced parental cells in a mixed culture. In parallel co-cultures, we compared the growth of control shRNA-transduced cells with parental non-transduced cells. For both of the Sulf-2 specific shRNAs, the non-silenced parental L3.6sl cells progressively overtook the silenced cells with time in culture. Similar results were seen with the BxPC-3 cells (Figure 5D). In contrast, there was no change in the ratio of the control-transduced cells to parental cells over time (Figure 5C).

### Silencing of Sulf-2 inhibits tumor growth in vivo

**Figure 6:**
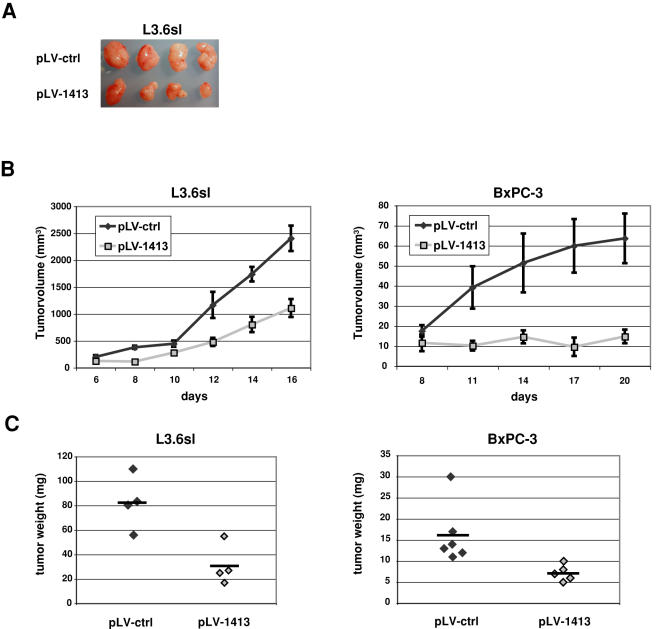
Tumorigenicity of L3.6sl and BxPC-3 cells silenced for Sulf-2. L3.6sl and BxPC-3 cells were transduced with either pLV-1413 or pLV-ctrl and subcutaneously injected into nude mice at 5×10^6^ per site. A: L3.6sl derived tumors harvested from mice after 17 days of growth B: Tumor size was monitored over time and values represent the mean (+/-SEM) of 4 mice for L3.6sl cells and 6 (pLV-ctrl) or 5 (pLV-1413) mice for BxPC-3 cells. C: Animals were sacrificed and tumor weight was examined. The difference in tumor weights between the two groups was significant for L3.6sl cells with a p-value of 0.01 and for BxPC-3 cells with a p-value of 0.029 (Student t-test).

To determine the effects of Sulf-2 silencing on the tumorigenicity of L3.6sl and BxPC-3 cells *in vivo*, we compared the growth of tumors that formed in immunocompromised mice with or without silencing of Sulf-2. Nude mice (CD-1) were injected subcutaneously with either pLV-ctrl or pLV-1413 transduced cells 1 week after transduction. Tumor size was followed with time by external palpation. At the end of the experiment, animals were sacrificed and tumor weights were measured. As shown in Figure 6, tumors derived from Sulf-2 silenced cells (L3.6sl or BxPC-3) grew at a markedly slower rate than those derived from the control cells (Figure 6B). The harvested tumors from silenced cells were correspondingly reduced in weight with no overlap of the two groups for either cell line (Figure 6C).

## Discussion

The prevailing view in the literature has been that the Sulfs are negative regulators of tumor cell growth. A number of reports demonstrated that over-expression of Sulf-1 [28]–[34] or Sulf-2 [32] in tumor cell lines inhibited growth factor signaling in response to several factors, including FGF-2, HB-EGF, and HGF. Moreover, the tumorigenicity of Sulf-overexpressing cells in immunocompromized mice was diminished [31], [32], [34]. The negative effects of Sulfs on FGF-2 signaling are consistent with the known requirement for 6S on HSPGs in the FGF-2 signaling complex [37]. These cell line findings have led to the suggestion that down-regulation of Sulfs could provide tumor cells with a growth advantage in certain settings. This model may have validity for the significant subset (two-thirds) of ovarian cancer cases [28] and the minor subset (29%) of hepatocellular carcinoma cases [30] in which *SULF1* expression is downregulated relative to that in normal tissue. However, the model is problematic for the sizeable subsets of several cancers which show increases in *SULF* expression. Thus, for example, *SULF1* is upregulated in significant subsets of breast cancer [38], pancreatic cancer [31], [39] (Table 1) lung adenocarcinoma [40] and hepatocellular carcinoma [30], while *SULF2* is upregulated in multiple myeloma [32], breast cancer and CNS cancers [41].

To understand the significance of upregulated *SULF* expression in a cancer, we chose to focus on pancreatic adenocarcinomas because of clear evidence associating this cancer with canonical Wnt signaling, a signaling pathway known to be positively regulated by the Sulfs during development (see above). Consistent with the transcript expression data (Table 1), we found that both Sulfs were strongly expressed at the protein level by malignant epithelial cells in pancreatic adenocarcinoma. To facilitate investigation of these enzymes in pancreatic cancer, we examined *SULF* expression in a large panel of pancreatic adenocarcinoma cell lines and found widespread expression, especially of *SULF2*. We chose 4 lines for study, all of which expressed Sulf-2 protein and one of which expressed both Sulfs. Previous studies have shown that proliferation and survival of 3 of these (BxPC-3, HS766T and L3.6sl) depend on activated Wnt signaling [M. Pasca di Magliano and M. Hebrok, unpublished observations]. We showed that Wnt signaling was markedly inhibited by overexpressing recombinant sFRP-2 in these cell lines. Moreover, overexpression of catalytically inactive Sulf-2 in the same lines resulted in a marked inhibition of Wnt activity, and exposure of the cells to inactive Sulf-2 protein during culture inhibited both Wnt signaling and growth. These results are consistent with Wnt-ligand dependent growth of these cells (“autocrine Wnt signaling”), which is modulated by the extracellular Sulfs. It is plausible that the CFPAC-1 cell line, which was refractory to all of these treatments, carries an activating mutation in downstream elements of the Wnt signaling pathway that would bypass the need for extracellular Wnt ligands and the Sulfs. It should be noted that the Sulfs represent an additional example of an extracellular factor, which modulates Wnt signaling. However, in contrast to the extracellular Wnt antagonists such as DKK1 and sFRPs, which are suppressed in certain tumors, the Sulfs are upregulated in pancreatic adenocarcinomas and exert a positive effect on Wnt signaling. Here we demonstrate that the Sulfs enhance the activity of the Wnt pathway in tumor cells, a function demonstrated previously only in a developmental context.

Our results indicate that catalytically inactive Sulf-2 can act as a dominant-negative mutant. There are several plausible mechanisms to explain this effect. One is that inactive Sulf-2 acts as a “substrate trapping mutant” such that it competes with the active enzyme for binding to heparan sulfate substrates, thereby blocking the ability of the enzyme to alter the substrate and modulate Wnt interactions. A second explanation is based on the observation that certain sulfatases (e.g., arylsulfatase A), can form homo-oligomers [42]. The Sulfs have the potential to form multimers due to their predicted coiled-coil structure [22]. Thus, the catalytically inactive protein could interfere with the general activity when incorporated into a multimer. Thirdly, the inactive Sulf might displace the wild-type enzyme from a subdomain in the plasma membrane where it is normally targeted for optimal function. The cell surface associations of QSulf-1 and QSulf -2 are known to require the hydrophilic domains of the molecules, but the molecular details have not been worked out [43].

To rigorously test the involvement of the Sulfs in Wnt signaling and tumor growth, we used lentiviral shRNA systems to silence Sulf-2 expression in four pancreatic adenocarcinoma cell lines. This approach contrasts with previous studies in which function of a Sulf was investigated in cancer cell lines by overexpressing it in cells that lacked it [28]–[34]. Sulf-2 silenced cells (BxPC-3, L3.6sl, HS766T) exhibited a decrease in DNA synthesis and an increase in apoptosis (BxPC-3, L3.6sl). Moreover, the silenced cells exhibited reduced Wnt activity by two criteria. Consistent with these findings, silenced cells showed a marked growth disadvantage in co-cultures with control cells. The dominant negative effects in the co-culture experiments with stably transfected HEK 293T cells (Figure 3C) suggest that Sulf-2 can function in *trans*, at least in the setting of artificial overexpression of the protein. Therefore, one might predict rescue of the silenced cells by the secreted Sulf-2 protein from control cells. We conclude that the rescue effect was at best partial, since the silenced cells grew at a slower rate than the control cells in the mixed culture.

The in vitro results were extended into a tumorigenesis model in nude mice by demonstrating that Sulf-2-silenced L3.6sl and BxPC-3 cells formed smaller tumors with time. Our evidence for the involvement of the Sulfs in the growth and tumorigenesis of pancreatic adenocarcinoma cells is most complete for Sulf-2. It remains to be seen what impact silencing Sulf-1 will have on a cell type that expresses this enzyme alone or in combination with Sulf-2 (e.g., HS766T cells). One might predict functional redundancy of the two enzymes based on the ability of either inactive Sulf to exert equivalent dominant negative effects (Fig. 3A). Moreover, the two enzymes exhibit indistinguishable enzymatic activities against heparin/heparan sulfate in vitro [22], [43], although there is recent genetic evidence for cooperative activities [26].

The present findings are the first to implicate the Sulfs as positive regulators of growth in a cancer, namely pancreatic tumors that are driven by autocrine Wnt signaling. Further xenograft experiments with cell lines and primary tumor cells, together with studies of mouse models of pancreatic adenocarcinoma [44], are needed to test this proposed role for the Sulfs. Accumulating evidence indicates that a number of other cancers also depend on autocrine Wnt signaling for growth [6]–[8], [15], [45]–[47]. Since *SULF* upregulation has been found in a number of these cancers, the potential role of these enzymes in enhancing tumorigenesis deserves exploration in these systems. As extracellular enzymes, the Sulfs would be very attractive therapeutics targets for both small molecule and antibody approaches.

## Materials and Methods

### Constructs

Sulf cDNA constructs were cloned in our lab as described [22]. For Sulf-2 siRNA experiments, we used the target region GCTGAAGCTGCATAAGTGC (1413) or GGAGTGGTGGTGTCAATAA (1143) and AACAGTCGCGTTTGCGACTGG as control. The hairpin oligonucleotides were cloned into the plasmids pSico or pLVTHM as described [48], [49]. To activate the shRNA in pSico, transduced BxPC-3 cells were sorted for GFP expression and subsequently transiently transfected with a cre recombinase plasmid to excise the CMV-GFP reporter cassette and thus activate expression of the shRNAs. 10 days after cre-recombinase transfection, cells were sorted for GFP negative cells and analyzed for HSulf-2 expression and used for further experiments. The pPGK-cre-bpa plasmid was generously provided by Dr. M.S. German and the pLVTHM was provided by Dr. S.S. Sidhu.

### Preparation of Anti-Sulf-1 Polyclonal Antibodies

Polyclonal antibodies were produced by ProSci. Rabbits were immunized and purified as described [25] with a peptide, derived from the predicted human Sulf-1 sequence (H1.5: CPKNLDVGNKDGGSYDLHRGO-COOH, where C denotes the cysteine residue added for coupling).

### Histology and immunoblotting assays

Under Committee for Human Experimentation approval (H1060-27616-01), archived formalin-fixed paraffin-embedded tissue blocks were obtained for seven cases of invasive pancreatic adenocarcinoma. Hydrated 4 µm sections were subject to heat induced epitope retrieval (10′ in a pressure cooker) and stained with either purified polyclonal rabbit Ab H1.5 or Ab H2.3. A purified polyclonal rabbit antibody directed to HSVI/II served as a negative control. Antibody binding was visualized by the Dako Envision (+) system (cat #K4011). Areas of invasive adenocarcinoma were identified by architectural features (infiltration and associated desmoplasia) and confirmed by cytologic features (nuclear enlargement, pleomorphism, and hyperchromasia) on H&E-stained sections. Interlobular ducts without any of the above features served as an internal control.

Cell lysates were prepared by extraction of washed cells in RIPA buffer (150 mM NaCl, 1% Triton X-100, 0.1% SDS, protease inhibitor cocktail (Roche), 50 mM Tris-HCl pH 7.5 for 20 min on ice. Insoluble material was pelleted by centrifugation for 30 minutes at 21.000 g. For analysis of conditioned medium (CM), the CM was concentrated on a Centricon30 microconcentrator (Millipore Corp.). For extracting nuclear proteins, we used the Compartmental Protein Extraction Kit (Chemicon International/Millipore). Equal protein concentrations were loaded based on quantification with the BCA™ Protein Assay (Pierce, Rockford, IL) following the manufacturer's procedure. Total cell lysates or CM were subjected to SDS-PAGE on 7.5 or 10% polyacrylamide gels (BioRad) blotted to ProBlott™ (Applied Biosystems). Immunoblotting was performed as described [22] with affinity purified rabbit polyclonal antibodies H1.5 for human Sulf-1 and H2.3 or H2.1 for human Sulf-2 [25]. For detection of β-catenin we used anti-active-β-catenin antibody (clone 8E7, Millipore), for histone detection we used anti-Histone H1 (clone AE-4, Millipore). For detection of actin we used the antibody “Actin (I-19)” from Santa Cruz Biotechnology Inc.

### Cell culture and gene transduction

Human pancreatic adenocarcinoma cell lines CFPAC-1, HS766T, L3.6sl and the human embryonic kidney cells HEK 293 were maintained in Dulbecco's modified Eagle's (DMEM) medium supplemented with 10% fetal bovine serum. Human pancreatic adenocarcinoma cell line BxPC-3 was maintained in RPMI medium supplemented with 10% fetal bovine serum. Sulf2 and S2ΔCC expressing HEK 293 cells were generated and grown as described [25]. We have previously verified the elimination of enzymatic activity in the S2ΔCC mutant [25]. We employed the same strategy for achieving stable expression of Sulf-1 and catalytically inactivated Sulf-1 in HEK 293 cells. For transient transfections, Fugene (Roche) or for Cre transfection the Basic Nucleofector Kit for Primary Mammalian Epithelial Cells (Amaxa) was used. Lentivirus production and infection was done as described [48], [49]. To generate pSico transduced cell lines 15–20 GFP positive colonies were picked and co-cultured. 10 days after Cre-transfection cell populations were sorted by FACS for GFP negative cells. NIH 3T3 fibroblast cells, expressing Wnts were generously provided by Dr. A. Kispert, Germany.

### RT-PCR and Data mining

For analyzing Sulf expression patterns in published gene microarray studies, we used the oncomine database (www.oncomine.org) and http://source.Stanford.edu. Total RNAs were extracted from cells using Trizol (Invitrogen) and cDNA was generated using Superscript II Reverse Transcriptase (Invitrogen). For RT-PCR the following primer combinations were used: Sulf-1 forward: 5′-CTCACAGTCCGGCAGAGCACGCGGAAC-3′, and reverse: 5′-CACGGCGTTGCTGCTATCTGCCAGCATCC-3′. For Sulf-2, the following primers were used: forward 5′-GAAAAGAGGCAGATTCACGTCGTTTCCAG-3′ and reverse 5′-ATCTGGTGCTTCTTTTGGGATGCGGGAG-3′. SYBR Green real time PCR was done, using the primer pairs: Sulf1F: 5′-TTGAAGAGAGCATAATTGGAATGG-3′, Sulf1R: 5′-CTGCATGTCATTTGCCA AGTTT-3′ and Sulf2F: 5′-C CTCTTCCCAAACGCATCTC-3′ and 5′-GCGCATGAT CCAGTGTTTGT-3′. The conditions for denaturing, annealing and extension of the template cDNA were as follows: 94°C for 30 sec, 55°C for 30 sec and 72°C for 1 min for 35 cycles. PCR products were separated by electrophoresis on 2% agarose gels and visualized with ethidium bromide.

### Luciferase reporter assays

Cells transfected with either TOPflash or FOPflash and Renilla-Luciferase control plasmid (Promega). After 48 hrs cells analyzed utilizing the Dual Luciferase Reporter Assay system (Promega). Luciferase activity was normalized to control Renilla-Luciferase activity. To determine Sulf influence on Wnt signaling in HEK 293T cells, cells were transfected with TOP/FOPflash and Renilla control plasmid and incubated with CM from Wnt1 or Wnt4 transfected 3T3 human fibroblasts for 24 hours.

### Co-culture experiment

Co-culture experiments were performed in transwell plates with 24 well inserts with 5 µm pore size (Corning Incorporated). HEK293T cells were plated in the wells as feeder layer and one day later 1–2×10^3^ cells of the pancreatic adenocarcinoma cell lines were plated in the inserts.

### BrdU incorporation assay

BrdU incorporation was measured by flow cytometry, using the APC BrdU Flow Kit (BD Biosciences), pulsing cells for one hour.

### Cell Apoptosis assay

Cell apoptosis was detected using the Annexin V PE apoptosis detection kit (BD Biosciences) following the manufacturer's protocol.

### Tumorigenicity assays:

BxPC-3 cells, transduced with either control or Sulf-2 shRNA were subcutaneously injected in 6 week nude mice at 5×10^6^ cells per site. Tumor growth was monitored every two to three days [7].

## Supporting Information

Figure S1 Sulf promotion of Wnt signaling in a reconstituted system. HEK 293T cells, stably transfected with either Sulf-1, Sulf-2 or a control vector, were co-cultured alone or with NIH 3T3 cells that were stably transfected with either Wnt1, Wnt2 or lacZ control. Wnt signaling in the various HEK 293T cells was measured by TCF-luciferase reporter activity. Values are normalized to control HEK 293T cells cultured alone.
(0.49 MB EPS)
